# Telomeric Double Strand Breaks in G1 Human Cells Facilitate Formation of 5′ C-Rich Overhangs and Recruitment of TERRA

**DOI:** 10.3389/fgene.2021.644803

**Published:** 2021-03-25

**Authors:** Christopher B. Nelson, Taghreed M. Alturki, Jared J. Luxton, Lynn E. Taylor, David G. Maranon, Keiko Muraki, John P. Murnane, Susan M. Bailey

**Affiliations:** ^1^Department of Environmental and Radiological Health Sciences, Colorado State University, Fort Collins, CO, United States; ^2^Cell and Molecular Biology Program, Colorado State University, Fort Collins, CO, United States; ^3^Department of Radiation Oncology, University of California, San Francisco, San Francisco, CA, United States

**Keywords:** telomeres, telomere double-strand breaks(DSBs), telomeric C-rich overhangs, telomere repeat-containing RNA (TERRA), alternative lengthening of telomeres (ALT), G1 human cells

## Abstract

Telomeres, repetitive nucleoprotein complexes that protect chromosomal termini and prevent them from activating inappropriate DNA damage responses (DDRs), shorten with cell division and thus with aging. Here, we characterized the human cellular response to targeted telomeric double-strand breaks (DSBs) in telomerase-positive and telomerase-independent alternative lengthening of telomere (ALT) cells, specifically in G1 phase. Telomeric DSBs in human G1 cells elicited early signatures of a DDR; however, localization of 53BP1, an important regulator of resection at broken ends, was not observed at telomeric break sites. Consistent with this finding and previously reported repression of classical non-homologous end-joining (c-NHEJ) at telomeres, evidence for c-NHEJ was also lacking. Likewise, no evidence of homologous recombination (HR)-dependent repair of telomeric DSBs in G1 was observed. Rather, and supportive of rapid truncation events, telomeric DSBs in G1 human cells facilitated formation of extensive tracks of resected 5′ C-rich telomeric single-stranded (ss)DNA, a previously proposed marker of the recombination-dependent ALT pathway. Indeed, induction of telomeric DSBs in human ALT cells resulted in significant increases in 5′ C-rich (ss)telomeric DNA in G1, which rather than RPA, was bound by the complementary telomeric RNA, TERRA, presumably to protect these exposed ends so that they persist into S/G2 for telomerase-mediated or HR-dependent elongation, while also circumventing conventional repair pathways. Results demonstrate the remarkable adaptability of telomeres, and thus they have important implications for persistent telomeric DNA damage in normal human G1/G0 cells (e.g., lymphocytes), as well as for therapeutically relevant targets to improve treatment of ALT-positive tumors.

## Introduction

Telomeres, specialized nucleoprotein complexes that “cap” the ends of linear chromosomes, are composed of highly conserved, G-rich tandem repeats [(5′-TTAGGG-3′)_*n*_ in vertebrates] (Meyne et al., 1989). Due to their repetitive nature and abundance of heterochromatic marks, telomeres were long regarded as silenced, non-transcribed features of the genome. Thus, the discovery of telomere repeat-containing RNA (TERRA) opened many new avenues of investigation (Azzalin et al., 2007). TERRA is a long non-coding RNA (lncRNA) that accumulates at telomeres, and contributes to structure and function via regulation of telomere length and maintenance of genome stability (Deng et al., 2009; Balk et al., 2013; Arora et al., 2014; Montero et al., 2018; Bettin et al., 2019).

Telomeres shorten with cell division (due to the end-replication problem) and thus with aging, as well as with a host of lifestyle factors, stresses, and environmental exposures. Telomeres end with a 3′ single-stranded (ss)G-rich overhang (Makarov et al., 1997) that serves as the substrate for telomerase (Greider and Blackburn, 1985). Telomerase is the specialized reverse transcriptase capable of maintaining telomere length via RNA template-mediated addition of telomeric repeats onto the ends of newly replicated chromosomes. Telomerase activity is sufficient only in highly proliferative populations like germ-line, stem, and the vast majority of cancer cells, thereby endowing them with extended or unlimited replicative potential (Kim et al., 1994; Batista, 2014). The remaining ∼10% of human cancers maintain telomere length via a recombination-dependent, alternative lengthening of telomere (ALT) mechanism (Bryan et al., 1997) that display a number of defining features, including heterogeneous telomere lengths, increased frequencies of telomere sister chromatid exchange (T-SCE), ALT-associated PML bodies (APBs), and extrachromosomal telomeric repeats (ECTR), which include C-rich (ss)circles (C-circles) (Murnane et al., 1994; Bailey et al., 2004; Henson et al., 2009; Cesare and Reddel, 2010). The ALT phenotype is relatively common in several subtypes of human sarcomas and astrocytomas, and has been observed in ∼4% of all tumor types, including carcinomas and pediatric glioblastoma multiformes (Heaphy et al., 2011).

Telomeric 3′ (ss)G-rich overhangs are also required for the formation of protective terminal structural features termed T-loops (Griffith et al., 1999; Tomaska et al., 2020). Telomeres are bound by shelterin, a six-member protein complex that includes the telomere-repeat binding factors TRF1 and TRF2, which contributes to regulation of telomerase activity, T-loop formation, and protection of chromosome ends (De Lange, 2005). Functional telomeres are essential for maintaining genome stability, as they protect natural chromosomal termini from degradation and prevent them from being recognized as double-strand breaks (DSBs) and triggering inappropriate DNA damage responses (DDRs) (De Lange, 2010; Baker et al., 2011; Sfeir and De Lange, 2012; De Lange, 2015). Inhibition of conventional repair activities at telomeres has also been demonstrated (Van Steensel et al., 1998; Bae and Baumann, 2007; Kibe et al., 2010; Sfeir and De Lange, 2012), raising the question of how—and even whether—DSBs occurring within telomeric repeats themselves are repaired. Various strategies employing targeted enzymatic cleavage of telomeric repeats have recently enabled studies to directly address this intriguing issue (Anzai et al., 2001; Yoshitake et al., 2010; Doksani and De Lange, 2016; Mao et al., 2016).

Enzymatically induced telomeric DSBs in murine cells have been shown to activate a DDR and recruitment of 53 binding protein 1 (53BP1) in a subpopulation of cycling cells, specifically those undergoing DNA replication (Doksani and De Lange, 2016). Moreover, homologous recombination (HR) and alternative non-homologous end-joining (alt-NHEJ), but not classical NHEJ (c-NHEJ), occurred following induction of telomeric DSBs in cycling cell populations (Doksani and De Lange, 2016; Mao et al., 2016). These results suggest that while repair of telomeric DSBs is possible, it may be limited to cells undergoing replication, i.e., involve elongation of broken telomeres. Such a notion is further supported by studies utilizing global DNA-damaging agents—ionizing radiation (IR) and hydrogen peroxide—which, although would only rarely be expected to directly produce telomere-specific DSBs, have demonstrated that telomeric damage responses persist in G1 cells that undergo senescence (Fumagalli et al., 2012; Hewitt et al., 2012). It is also true that due to their G-rich nature, telomeres are particularly susceptible to oxidative damage. Furthermore, chronic oxidative stress and persistent telomeric DSBs have been shown to activate the ALT pathway and/or induce ALT-like phenotypes (Coluzzi et al., 2017; Liu et al., 2018). Also of relevance to damaged or broken telomeres, short telomeres recruit TERRA via R-loops more efficiently than longer telomeres (Feretzaki et al., 2020).

Whether or not repair of telomeric DSBs requires cell cycle progression (replication) has physiological relevance, as many human adult tissues are largely post-mitotic (or quiescent), and unrepaired DSBs can trigger senescence, thereby contributing to degenerative pathologies (Fumagalli et al., 2012; Hewitt et al., 2012). Here, we investigated human cellular responses to targeted telomeric DSBs specifically in G1 phase, utilizing the previously characterized telomere-specific endonuclease TRAS1-EN-TRF1 (EN-T) (Anzai et al., 2001; Yoshitake et al., 2010) in telomerase-positive fibroblasts and cancer cells, and in telomerase-independent ALT cells. Signatures of an early DDR were observed, as both gamma (γ)-H2AX and mediator of DNA damage checkpoint protein 1 (MDC1) foci co-localized with broken telomeres. However, and consistent with previous reports, 53BP1 did not significantly overlap with telomeric DSBs in G1 (Doksani and De Lange, 2016).

Due to the scarcity of a homologous template, NHEJ is regarded as the primary DSB repair pathway during G1 in mammalian cells (Lieber, 2010; Chapman et al., 2012; Chang et al., 2017). Consistent with inhibition of c-NHEJ at telomeres (Miller et al., 2011; Muraki et al., 2013), we show with both short hairpin (sh)RNA depletion and chemical inhibition of the key NHEJ kinase, DNA-dependent protein kinase catalytic subunit (DNA-PKcs), that c-NHEJ is not a major contributor to repair of telomeric DSBs in G1 human cells. Furthermore, and consistent with lack of RAD51 in G1 (Tashiro et al., 1996), no evidence of classical HR-dependent repair of telomeric DSBs in G1 was found, as neither RAD51, RAD52 (early responders that promote and stimulate strand invasion, respectively), nor repair-associated DNA synthesis (BrdU incorporation) were detected. The most striking observation at telomeric DSBs in G1 were extensive tracks of predominantly 5’ C-rich (ss)telomeric DNA, which co-localized with replication protein A (RPA) in telomerase-positive human cells. Consistent with this finding, S4/S8 phosphorylated RPA (pRPA) foci, which are associated with activation of RPA during DNA repair (Maréchal and Zou, 2015), had only modest dependence on the conventional end processing exonucleases MRE11 (3′-to-5′) and EXO1 (5′-to-3′). The 5′-to-3′ nuclease Apollo, which has been implicated in post-replicative processing specifically of leading-strand telomeres (Lam et al., 2010), also did not influence resection at telomeric DSBs in G1.

These results support the view that telomeric DSBs in G1 human cells represent rapid truncation events, in that they facilitate formation of 5′ C-rich (ss)overhangs, previously proposed markers of the recombination and replication-dependent ALT pathway of telomere maintenance (Oganesian and Karlseder, 2011). Indeed, induction of telomeric DSBs in human U2OS (ALT, osteosarcoma) cells resulted in significant increases in 5′ C-rich (ss)telomeric DNA in G1, which was bound by the complementary telomeric RNA, TERRA. We propose that enrichment of 5′ C-rich (ss)telomeric DNA in G1 results from telomere DSB-mediated deletion of protective T-loops and extensive resection in the absence of 53BP1. Furthermore, these exposed and vulnerable structures are promptly protected by interactions involving either RPA or transient telomeric RNA:DNA hybrids (Ohle et al., 2016; Feretzaki et al., 2020) dependent on telomerase status, presumably allowing them to persist into S/G2 for telomerase-mediated replication or HR-dependent elongation, while also circumventing conventional repair pathways (D’Alessandro et al., 2018).

## Materials and Methods

### Cell Culture and Transfections

Human U2OS (ALT), U2OS RAD52-YFP (obtained from Jiri Lucas, University of Copenhagen), and EJ-30 cancer (obtained from Dr. John Murnane, UCSF) cells were cultured in Dulbecco’s modified Eagle medium (DMEM, Hyclone) supplemented with 10% fetal bovine serum (FBS). Telomerase-positive, apparently normal human BJ1 hTERT fibroblasts (ATCC) were cultured in Alpha-MEM (Hyclone) supplemented with 10% FBS.

TRAS1-EN-TRF1 and TRF1 plasmids, both driven by a CMV promoter and possessing a C-terminal Flag tag for visualization, were constructed from a CMV-driven TRAS1-EN-TRF1 plasmid obtained from Dr. Haruhiko Fujiwara (University of Tokyo). Transient transfections were carried out with Lipofectamine 3000 (Invitrogen) at 60–80% confluency in Opti-MEM (Gibco) for 20 min and replaced with normal media 8 h later. Unless otherwise specified, all experiments were carried out 48 h post transfection.

A U2OS cell line stably expressing FUCCI-Germinin (Green; S, G2/M) was established by transfecting cells with 0.5 μg of Kan-FUCCI-Green (S-G2-M) plasmid. Plasmids were delivered using Lipofectamine 3000 (Invitrogen) following the manufacturer’s instructions. Eight hours following transfection, Opti-MEM media was replaced with fresh DMEM media. One week later, cells were trypsinized and individual cells were seeded in a 96-well plate. A positive single clone was identified, and expanded in the presence of 800 μg/ml of G-418 sulfate (GoldBio). After reaching 90% confluency, cells were split into a 24-well plate, 10 days later into a 12-well plate, and 1 week after into a six-well plate. Cells were transferred into a T-25 flask and then into a T-75 flask after 6 days. The DMEM media containing 800 μg/ml of G-418 sulfate was changed every 2 days.

### Laser Micro-Irradiation

Laser micro-irradiations were performed with a Zeiss LSM880 confocal microscope using a 405-nm laser at 100% with settings of 50 iterations and a 15 s pixel dwell. Spatially defined stripes of damage were generated through nuclei of cells followed by a recovery period of 30 min. Immunofluorescence and imaging of micro-irradiated cells were carried out as for other experiments and as described below.

### RNA Interference

Small interfering (si)RNA was delivered into cells using Lipofectamine RNAiMAX in OptiMEM media according to the manufacturer’s instructions (ThermoFisher), followed by replacement with regular media 5 h later. Twenty-four hours following initial siRNA delivery, cells were co-transfected with EN-T or TRF1-only and appropriate siRNA in Lipofectamine 3000 according to manufacturer’s instructions (ThermoFisher), and then fixed or harvested 48 h later. siRNA sequences were: TRF2 5′-GAGGAUGAACUGUUUCAAGdtdt-3′ (anti-sense also included 3′ dtdt) and EXO1 5′-UGCCUUUGCUAAUCCAAUCCCACGC-3′. A stable DNA-PKcs-deficient BJ1-hTERT cell line was generated using MISSION lentiviral transduction particles (Sigma-Aldrich) and 1 μM puromycin selection. Short hairpin (sh)DNA-PKcs sequences were: 5′-CCGGCCAGTGAAAGTC TGAATCATTCTCGAGAATGATTCAGACTTTCACTGGTTTT T-3′ and 5′-CCGGCCTGAAGTCTTTACAACATATCTCGAGA TATGTTGTAAAGACTTCAGGTTTTTTG-3′.

### Inhibitors

BJ1-hTERT fibroblasts were treated with either a specific DNA-PKcs kinase inhibitor that prevents autophosphorylation (NU7026, Sigma), or a MRE11 endonuclease activity inhibitor (PFM01, ThermoFisher). NU7026 was used at a concentration of 10 μM for 24 h prior to harvesting cells as per previous (Le et al., 2013). Alternatively, PFM01 was used at a concentration of 100 μM for 8 h preceding fixation. For chromatin relaxation, cells were treated with trichostatin A (TSA, Sigma) at the specified concentrations for 24 h prior to cell fixation.

### Western Blotting

Cell pellets were washed in phosphate-buffered saline (PBS) and then incubated in lysis buffer for 10 min. Lysis buffer consisted of mammalian protein extraction reagent (M-PER, ThermoFisher) with protease inhibitors (complete mini EDTA free, Sigma-Aldrich), and in cases when phosphorylated proteins were being detected, phosphatase inhibitors (PhosSTOP, Sigma-Alrdrich). Following isolation of protein, the Bradford assay was used to quantify protein (BioRad). Thirty micrograms of protein was loaded into precast SDS–PAGE gels (Mini-Protean TGX, 4-15%, BioRad) in Tris/Glycine/SDS buffer followed by electrophoretic separation for roughly 1.5 h at 125 V. After electrophoresis, proteins were transferred to a polyvinylidene fluoride (PVDF) membrane in Tris/Glycine buffer with 10–15% methanol for 16–20 h at 30 V at 4°C. An even protein transfer was verified by reversibly staining membranes with Poncaeu S solution (Sigma-Aldrich, 0.1% w/v in 1% acetic acid). Next, membranes were blocked in 5% non-fat dry milk (NFDM) or bovine serum albumin (BSA) in 1X Tris buffered saline with 0.1% Tween 20 (TBST) from 30 min to 1 h with gentle shaking. Blocking solution was then replaced with fresh blocking solution containing the appropriate dilution of primary antibody and incubated from 2 h to overnight with gentle shaking. Following primary antibody incubation, membranes were washed in 1X TBST for four washes of 10 min each with gentle shaking. Next, fresh blocking solution was added with the appropriate dilution of a horseradish peroxidase (HRP)-labeled secondary antibody and incubated from 2 to 4 h followed by another series of four washes in 1X TBST. Following the final wash, membranes were rinsed in PBS. To visualize proteins, membranes were treated with SuperSignal^TM^ West Pico Chemiluminescent Substrate according to the manufacturer’s instructions (ThermoFisher) and imaged on a ChemiDoc^TM^ XRS + imager with ImageLab software (BioRad).

Primary antibodies for Western blotting included Rabbit Anti-phospho serine2056 DNA-PKcs (Abcam ab1249181, 1:2,000), Mouse Anti-DNA-PKcs (ThermoFisher MS-423-P, 1:10,000), Mouse Anti-TRF2 (SantaCruz sc-271710, 1:500), Mouse Anti-phospho serine1981 ATM (Upstate 05–740, 1:1,000), Rabbit Anti-phospho Thr68 CHK2 (Cell signaling 2,661, 1:1,000), Rabbit Anti-EXO1 (Proteintech 16352-1-AP, 1:500). HRP-labeled secondary antibodies included Donkey Anti-Rabbit (Jackson ImmunoResearch 711-035-152, 1:20,000) and Rabbit Anti-Mouse (ThermoFisher 816720, 1:10,000).

### Immunofluorescence

Unless stated otherwise, cells were grown on chamber slides, rinsed twice in PBS, fixed in freshly prepared 4% paraformaldehyde (PFA) for 10 min at room temperature, and then permeabilized in 0.2% Triton X-100 in PBS for 4–10 min. Next, cells were blocked in 10% normal goat serum (NGS) or 5% BSA in 1 × PBS for 40 min and then incubated with primary antibodies diluted in blocking solution for 1 h at 37°C or overnight at 4°C. Following primary incubations, cells were washed three times in 1 × PBS at 42°C. After washes, cells were incubated with fluorophore-conjugated goat secondary antibodies for 30 min at 37°C. Finally, cells were washed again as before and counterstained with Prolong Gold Antifade reagent with DAPI (Invitrogen).

Primary antibodies and concentrations included Rabbit Anti-53BP1 (Bethyl A300-272A, 1:800), Rabbit Anti-γ-H2AX (Bethyl A300-081, 1:1,000), Mouse Anti-Flag (Sigma M2 F1804, 1:2,000–4,000), Rabbit Anti-RPA70 (Cell signaling #2267, 1:50), Rabbit Anti-phospho S4/S8 RPA32 (Bethyl A300-245A 1:2,000), Mouse Anti- γ-H2AX (Millipore 05-636, 1:1,500), Rabbit Anti-Cyclin A (Santa Cruz SC-751, 1:500), Rabbit Anti-MDC1 (Bethyl A300-051A, 1:1,000), Rabbit Anti-RAD51 (H-92 SC-8349, 1:800), Sheep Anti-RAD52 (kind gift from Jiri Lukas Lab, 1:100), Rat anti-BrdU (BioRad OBT0030, 1:200), and Rabbit Anti-phospho S15 53 (Abcam Ab18128-50, 1:500).

Secondary antibodies and concentrations included Alexa-488 Goat anti-Mouse (ThermoFisher A11029, 1:750), Alexa-594 Goat anti-Mouse (ThermoFisher A11005, 1:750), Alexa-647 Goat anti-Mouse (ThermoFisher A21235, 1:350), Alexa-488 Donkey anti-Mouse (ThermoFisher 21202, 1:750), Alexa-488 Goat anti-Rabbit (ThermoFisher A11008, 1:750), Alexa-594 Goat anti-Rabbit (ThermoFisher A11012, 1:750), Alexa-555 Goat anti-Rat (ThermoFisher A21434, 1:750), Alexa-647 Donkey anti-Sheep (ThermoFisher A21448, 1:350), and Alexa-488 Donkey anti-Mouse (ThermoFisher A21202 1:750).

### BrdU Incorporation Assay

Cells were pulse-labeled with the thymidine analog Bromodeoxyuridine/5-bromo-2′-deoxyuridine (BrdU; ThermoFisher) for 2 h (50 mM) and then fixed for 15 min in 4% PFA at room temperature. Next, cells were permeabilized for 20 min with 0.1% Triton x-100 in PBS, followed by DNA denaturation for 10 min on ice with 1 N HCl and then 10 min at room temperature with 2 N HCl. Cells were then washed with phosphate citric acid buffer pH 7.4 for 10 min at room temperature. Finally, cells were washed for 5 min in permeabilization solution. Blocking was then carried out for 30 min at 37°C in 5% NGS with 0.1% Triton X-100 in PBS. Antibody incubations, washing steps, and counterstaining were carried out as described for immunofluorescence.

### Non-Denaturing Immuno-Fluorescence *in situ* Hybridization

Combined immunofluorescence and fluorescence *in situ* hybridization (FISH) experiments were carried out on cells grown on chamber slides. Cells were initially fixed in 4% PFA for 5 min at room temperature. Next, cells were permeabilized for 4 min in 0.2% Triton X-100 in PBS. Following permeabilization, cells were blocked and immunostained as described in the immunofluorescence section. After the last washing step, cells were post-fixed in 4% PFA for 15 min at room temperature. Next, cells were dehydrated in an ethanol series (75, 85, and 95%) for 2 min each and allowed to air dry. While slides were air drying, the hybridization solution was prepared by combining 36 μl of formamide, 12 μl 0.05 M Tris–HCL, 2.5 μl 0.1 M KCL, 0.6 μl 0.1 M MgCl_2_, and 0.5 μl of 0.5 μM peptide nucleic acid (PNA) telomere probe (TelC-Alexa488 or TelG-Cy3, Biosynthesis) in 20% acetic acid. Hybridization solution was then denatured at 85°C for 10 min followed by cooling on ice. After cooling, 50 μl of hybridization solution was added to each slide, then slides were coverslipped, and incubated at 37°C in a humidified chamber for 6 h. Following hybridization, coverslips were removed and slides washed twice in 50% formamide in 2X SSC (2.5 min at 42°C), twice in 2X SSC (2.5 min at 42°C) and twice in 2X SSC + 0.1% NP-40 (2.5 min at 42°C). Following the final wash, cells were counterstained with Prolong Gold Antifade with DAPI.

### Fluorescence Microscopy and Image Analysis

Images were acquired using a Zeiss Axio Imager.Z2 epi-fluorescent microscope using a 63X/1.4 N.A. objective (Plan-APOCHROMAT, Zeiss). For the majority of targets, images were blindly and subjectively thresholded and segmented followed by determination of foci overlap (50% overlap scored as positive) in Metamorph 7.7 (Molecular Devices). For RAD52-YFP, RPA, and phospho-RPA foci analysis, cells tended to have very few or an abundance of foci, and scoring was therefore done on the basis of whether a cell had > 4 foci overlapping Flag.

Analysis of the laser microirradiation experiment involved first thresholding TRF2 foci using a fixed value for all images. Next, these thresholded foci were converted to regions in Metamorph and these regions transferred to γ-H2AX or 53BP1 images. Next, the average intensity within the transferred regions was compared with that within pseudo-random regions of comparable dimensions generated by rotating TRF2 images by 90°.

For BrdU foci analysis in BJ1-hTERTs, untransfected S-phase cells were excluded from analysis, which were identified by very bright pan nuclear staining. For DAPI-intensity-based cell cycle analysis, DAPI intensity was collected alongside foci by subjective thresholding and segmentation in Metamorph followed by histogram generation. Foci counts were sorted based on whether the nuclear intensity fell into the clear G1 peak or the S/G2 tail. The border region between cell cycle phases of four DAPI intensity bins was excluded from analysis to ensure accurate classification of cells.

### Telomere Restriction Fragment Southern Blots

The TRF assay was performed using the TeloTAGGG^TM^ Southern blotting kit (Roche) according to the manufacturer’s instructions with some modifications, including a longer probe hybridization time (6 h), as well as a longer incubation time with Anti-DIG antibody (1 h). Two micrograms of sample DNA were loaded per lane, and blots were imaged on a ChemiDoc^TM^ XRS + imager with ImageLab^TM^ software (BioRad). Quantitation of mean TRF length was performed using TeloTool software according to the manufacturer’s protocol.

### Statistical Analyses

EN-T validation experiments in U20S cells were done in duplicate (50 cells per replicate). Experiments with BJ1-hTERT cells involved three independent experiments for each condition with at least 30 cells per replicate for imaging experiments unless otherwise stated. The micro-irradiation experiments were done in five to seven cells per staining condition across a minimum of 50 telomeres (foci). Experiments with EJ-30 cells were also done in triplicate; however, the number of cells imaged totaled at least 300 per condition to allow for DAPI intensity histogram generation.

Error bars on bar graphs represent standard error of the mean (SEM); p-values were computed when experiments were done in triplicate. For each analysis, ANOVAs were performed to determine significance, followed by *post hoc* Tukey’s HSD where *p* < 0.05 was considered significant. ANOVAs were either one way or two way depending on the number of categorical independent variables.

## Results

### Characterization of Targeted Telomeric Double-Strand Breaks in G1 Human Cells

To better understand human cellular responses to telomeric DSBs throughout the cell cycle, we performed transient transfection experiments using a previously characterized plasmid encoding a flag-tagged telomere repeat-specific endonuclease fused to the human TRF1 gene (TRAS1-EN-TRF1: hereafter referred to as EN-T) that produces blunt-ended ended DSBs within telomeres (Anzai et al., 2001; Yoshitake et al., 2010). Several human cell lines with different telomerase status were selected. U2OS (ALT, telomerase independent) osteosarcoma cells served as a positive control for EN-T activity as cycling ALT cells undergo recombinational repair at telomeric DSBs. BJ1-hTERT-immortalized fibroblasts (telomerase positive) represent an apparently normal, non-tumorigenic human cell line. EJ-30, a bladder carcinoma cell line (highly telomerase positive) was also employed, as they have been used extensively to study sub-telomeric DSB repair (Zschenker et al., 2009; Miller et al., 2011; Muraki et al., 2013).

Following transient transfection with EN-T or TRF1-only, co-localization with telomeres was observed in all cell lines (Supplementary Figure 1A). Evidence of DSB signaling following EN-T expression in EJ-30 cells was also evaluated, which included phosphorylation of ATM (S1981) and CHK2 (Thr68) (Supplementary Figure 1B). Consistent with previous reports of TRF1 overexpression inducing DSBs [via a different mechanism involving telomere association, anaphase bridges and breakage (Van Steensel and De Lange, 1997; Palm and De Lange, 2008; Munoz et al., 2009)], TRF1-only also induced DSB signaling activity (less than EN-T), noted by the increase in intensity of phospho-ATM and phospho-CHK2 bands in transfected samples relative to no treatment controls. Supportive of telomere-specific cutting and rapid truncation events with EN-T, a decrease in telomere restriction fragment (TRF) size was also observed following EN-T expression, compared with TRF1-only and untransfected controls (Supplementary Figures 1C,D). EN-T expression reduced the mean TRF by ∼5–10%, consistent with expectation considering the relatively low transfection efficiency in EJ-30 cells (∼20–30%) and that not all telomeres were broken (∼8–12/cell visualized).

To further validate the EN-T system, we sought to reproduce the finding that induced telomeric DSBs stimulate a damage response and repair via some combination of HR and break-induced replication (BIR) in cycling ALT cells (Cho et al., 2014; Dilley et al., 2016). Human U2OS (ALT) cells exhibited activation of telomere damage responses upon transfection with EN-T as expected, and as evidenced by increased γ-H2AX foci compared with untransfected controls (Supplementary Figure 2A). Importantly, a significant portion of these well-accepted DSB damage markers occurred at broken telomeres, as γ-H2AX foci co-localized with EN-T foci. Additionally, following EN-T transfection, cycling U20S cells harbored elevated numbers of RAD51- and RAD52-YFP foci, mediators of HR and BIR respectively, which frequently co-localized with EN-T foci, confirming DDR/repair of telomeric DSBs by HR and BIR in *cycling* human ALT cells (Supplementary Figures 2B,C).

As various lines of evidence have suggested that DSB repair may be non-conventional or even non-existent within or near telomeres in G1 (Fumagalli et al., 2012; Hewitt et al., 2012; Doksani and De Lange, 2016; Mao et al., 2016), we sought to investigate DDRs and repair of broken human telomeres in G1 directly. In order to study telomeric DSBs in non-ALT EJ-30 (telomerase positive; cancer) cells specifically in G1, we employed a DAPI intensity-based approach as a means of distinguishing cell cycle phases in interphase nuclei, which retained the ability to make accurate measurements of fluorescent foci. Cells in G1 form a clear peak in the lower intensity portion of a DAPI intensity histogram using even a relatively low number (∼300) of cells (Supplementary Figure 3A). The specificity of the G1 DAPI intensity peak was validated via exclusion of Cyclin A, which stains S and G2 cells (Supplementary Figure 3B). A similar DAPI intensity histogram was generated to distinguish G1 from S/G2 in all imaging experiments involving EJ-30 cells. Reliable discrimination between S and G2 cells was not possible, therefore these populations were pooled throughout the analyses.

Although transfection efficiencies in BJ1-hTERT fibroblasts were quite low (0.5–2%), telomeric DSB induction was evaluated in EN-T and TRF1-only transfected cells. Consistent with expectation, only a very small percentage of transfected cells stained positive for Cyclin A (EN-T: 0%, TRF1: 3.2%, Supplementary Figure 3B) or incorporation of bromodeoxyuridine (BrdU; Supplementary Figure 3C), confirming that the vast majority of transfected BJ1-hTERT cells were in G1 phase 48 h post transfection, when analyses were performed. Transfection efficiencies were much higher (∼25–30%) in U2OS (ALT) cells (EN-T, TRF1-only, empty vector). A stably transfected fluorescent ubiquitination-based cell cycle indicator (FUCCI) (Sakaue-Sawano et al., 2008) U2OS cell line was also generated to definitively identify cell cycle phase, so for these EN-T experiments, scoring was restricted to only transfected cells (100%).

### Non-Canonical DNA Damage Response at Telomeric Double-Strand Breaks in G1 Human Cells Lacks Recruitment of 53 Binding Protein 1

**FIGURE 1:**
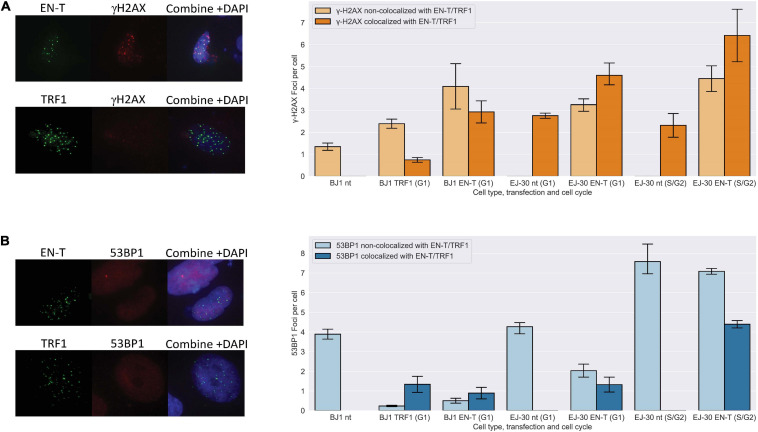
Telomeric double-strand breaks (DSBs) co-localize with γ-H2AX, but do not recruit 53 binding protein 1 (53BP1) to break sites in G1. **(A)** Transfection with EN-T resulted in co-localization of γ-H2AX foci with EN-T in BJ1-hTERT G1 cells (*p* = f0.009), and in EJ-30 G1 and S/G2 cells (*p* = 0.0009 and 0.022, respectively). **(B)** However, 53BP1 foci did not significantly co-localize with EN-T induced telomeric DSBs in BJ1 hTERT or EJ-30 G1 cells; 53BP1 foci only significantly co-localized at EN-T induced telomeric DSBs in S/G2 EJ-30 cells (*p* = 0.012). For TRF1-only, γ-H2AX occasionally co-localized with TRF1 foci in G1 cells, but overall were not significantly increased (*p* = 0.511), and 53BP1 foci were reduced compared to non- transfected (nt) control (*p* = 0.026). All data represent three independent experiments (*n* = 30 BJ1 hTERT; *n* = 300 EJ-30 cells/experiment). Error bars are standard error of the mean (SEM), significance was established using ANOVA and *post hoc* Tukey’s HSD; *p* < 0.05 is significant.

I-SCE1 induced DSBs in sub-telomeric regions have previously been shown to be refractory to repair (Miller et al., 2011; Muraki et al., 2013). To investigate damage responses at DSBs within individual telomeres, we evaluated co-localization of γ-H2AX and 53BP1 foci at EN-T induced telomeric DSBs. Telomeric DSBs (EN-T) co-localized with γ-H2AX in BJ1-hTERT G1 cells (*p* = 0.009) and in all phases of the cell cycle in EJ-30 cells (*p* = 0.0009 in G1 cells; *p* = 0.022 in S/G2 cells) (Figure 1A). Telomeric DSBs (EN-T) also co-localized with 53BP1 in EJ-30 S/G2 cells (*p* = 0.012). However, 53BP1 foci showed only minimal overlap with telomeric DSBs in BJ1-hTERT (*p* = 0.019) and EJ-30 (*p* = 0.062) G1 cells (Figure 1B). 53BP1 foci were reduced compared with the nt control (*p* = 0.026), and consistent with TRF1-only inducing some degree of telomeric damage, γ-H2AX occasionally co-localized with TRF1 foci in G1 cells, but overall were not significantly increased (*p* = 0.511) (Figures 1A,B).

**FIGURE 2:**
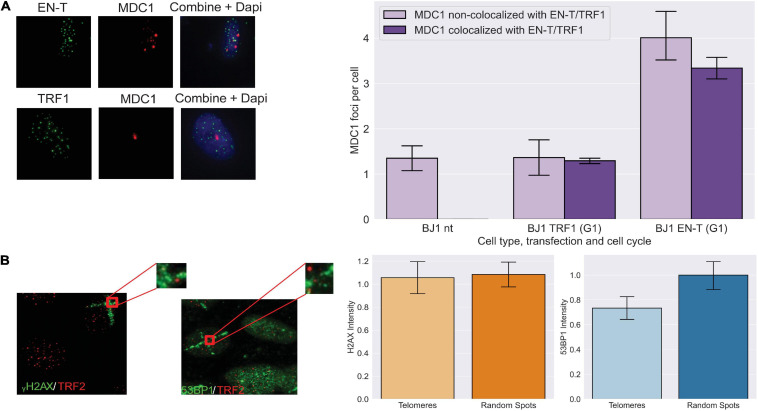
Early DNA damage response at telomeric DSBs in G1. **(A)** Transfection with EN-T resulted in increased numbers of mediators of DNA damage checkpoint protein 1 (MDC1) foci in BJ1-hTERT G1 cells (compared to non-transfected and TRF1 control cells), which often co-localized with EN-T (*p* < 0.0001). **(B)** The intensity of γ-H2AX within microirradiation-induced DNA damage stripes was similar at telomeres and random spots in non-transfected BJ1 hTERT cells, while the intensity of 53BP1 within damage stripes was decreased at telomeres relative to random spots (*p* = 0.099). All data represent three independent experiments (*n* = 30). Error bars are SEM, significance was established using ANOVA and *post hoc* Tukey’s HSD; *p* < 0.05 is significant.

To determine whether other components of a DDR were activated by telomeric DSBs in G1, we evaluated MDC1, an early mediator of the response to genomic DSBs that acts downstream of γ-H2AX, but upstream of 53BP1 (Stewart et al., 2003). MDC1 foci were significantly induced (*p* < 0.0001) and to a similar degree as γ-H2AX in response to telomeric DSBs in BJ1-hTERT G1 cells (Figure 2A). To evaluate whether other DNA-damaging methodologies might also initiate a DDR lacking 53BP1 recruitment to broken telomeres, we compared the intensities of γ-H2AX and 53BP1 foci co-localized at telomeres versus at random genomic sites that occurred within spatially defined stripes of damage generated by laser microirradiation. Although on average, cells with 53BP1 stripes had 7.1 telomeres, and cells with γ-H2AX stripes had 12.9 telomeres within the damage stripe, stripe size varied considerably. Therefore, telomeres not colocalizing with the DDR marker within the stripes were evaluated. Consistent with our results using EN-T, 30 min after exposure of BJ1-hTERT cells, the intensity of γ-H2AX was found to be similar at telomeres and random sites within the microirradiation stripe, while the intensity of 53BP1 was reduced at telomeres compared with random sites (*p* = 0.099; Figure 2B).

Last, we reasoned that normal telomere protection might prevent recruitment of 53BP1 to broken telomeres. Therefore, a variety of strategies were employed to compromise telomeric end-capping, including relaxation of chromatin utilizing the histone deacetylase inhibitor Trichostatin A (or exposure to a hypotonic solution; not shown), partial depletion of the shelterin component TRF2 via small interfering (si)RNA knockdown (above the level that induces a damage response), as well as small hairpin (sh)RNA knockdown of shelterin-associated DNA-PKcs (Supplementary Figures 4A–D). However, none of these conditions resulted in recruitment of 53BP1 to telomeric DSBs in G1 human cells. Together, these results further support the finding that although telomeric DSBs in G1 activate an early DDR (γ-H2AX and MDC1 recruitment), they do not attract 53BP1 to telomeric break sites.

### Classical Non-Homologous End-Joining Does Not Significantly Contribute to Repair of Telomeric Double-Strand Breaks in G1 Human Cells

**FIGURE 3:**
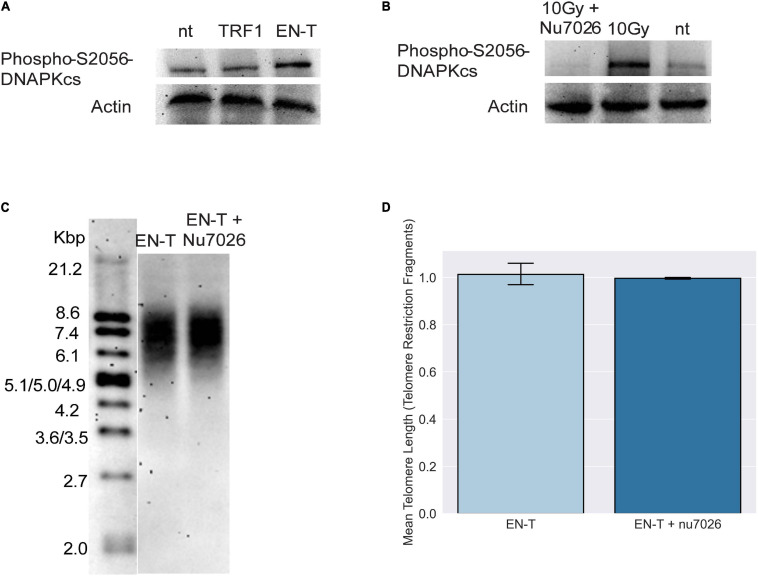
Consistent with absence of 53BP1, evidence of classical non-homologous end-joining (c-NHEJ) at telomeric DSBs was lacking. **(A)** Autophosphorylation of DNA-dependent protein kinase catalytic subunit (DNA-PKcs) at S2056 was induced following EN-T transfection of EJ-30 cells. **(B)** DNA-PKcs autophosphorylation following exposure to 10 Gy ionizing radiation (gamma rays) was prevented by the specific kinase inhibitor NU7026. **(C)** To assess the role of c-NHEJ specifically at broken telomeres, EJ-30 cells transfected with EN-T were treated with NU7026, which did not significantly influence mean telomere length (TRFs) compared with untreated control. **(D)** TRF quantification. Error bars are SEM, significance was established using ANOVA and *post hoc* Tukey’s HSD; *p* < 0.05 is significant.

The absence of 53BP1 at telomeric DSBs in G1, particularly with shRNA knockdown of DNA-PKcs, suggested that consistent with previous reports (Dimitrova et al., 2008; Zimmermann et al., 2013; Xiong et al., 2015), c-NHEJ may not be occurring at EN-T induced broken telomeres. Autophosphorylation of DNA-PKcs at serine 2056 was slightly increased in EJ-30 cells expressing EN-T compared with cells expressing TRF1-only or no treatment controls, and ionizing radiation (IR)-induced DNA-PKcs autophosphorylation was prevented by treatment with the specific kinase inhibitor NU7026 (Figure 3A). Considering that EN-T produced the most telomere-specific damage (Figure 1), we tested whether DNA-PKcs autophosphorylation influenced telomere DSB repair by comparing TRFs in cells expressing EN-T with those expressing EN-T and treated with NU7026 (Figure 3B). Chemical inhibition of DNA-PKcs autophosphorylation (NU7026, 24 h) in cycling EJ-30 cells expressing EN-T did not change the TRF size relative to the non-treated control, supporting the supposition that c-NHEJ does not significantly contribute to repair of telomeric DSBs (Figures 3C,D).

### Telomere-Specific Double-Strand Breaks in G1 Are Characterized by 5′ C-Rich (ss)Telomeric DNA

**FIGURE 4:**
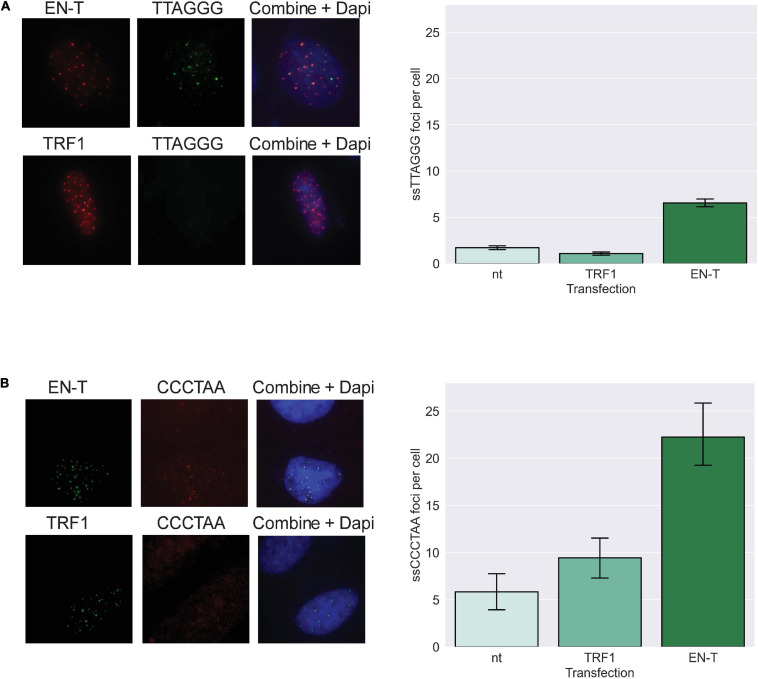
Extensive resection at telomeric DSBs in G1 facilitates formation of 5′ C-rich (ss)telomeric DNA. **(A)** Transfection of BJ1-hTERT cells with EN-T promoted modest production of G-rich (5′-TTAGGG-3′) (ss)telomeric DNA in G1 (*p* = 0.0002), and **(B)** significantly higher frequencies of C-rich (5′-CCCTAA-3′) (ss)telomeric DNA (*p* = 0.045). All data represent three independent experiments (*n* = 30). Error bars are SEM, significance was established using ANOVA and *post hoc* Tukey’s HSD; *p* < 0.05 is significant.

We further hypothesized that telomeric DSBs that fail to recruit 53BP1 may be particularly vulnerable to resection. To investigate the presence of ssDNA at telomeric DSBs in G1, fluorescence *in situ* hybridization (FISH) using a C-rich telomere probe—without denaturation of the DNA duplex [to detect 3′ G-rich (ss)telomeric DNA]—was performed in BJ1-hTERT G1 cells transfected with EN-T or TRF1-only. Indeed, telomeric ssDNA was more abundant in cells transfected with EN-T compared with TRF1-only or no treatment controls (*p* = 0.0002, Figure 4A). To determine whether resection occurred bidirectionally, we also performed the ssFISH assay with a G-rich telomere probe [to detect 5′ C-rich (ss)telomeric DNA]. Interestingly, hybridization with the G-rich probe produced many more signals overall, and more signals in EN-T than in TRF1-only transfected cells or no treatment controls (*p* = 0.045, Figure 4B). These results reveal that the telomeric ssDNA present at telomere-specific DSBs in G1 is enriched for 5′ C-rich (ss)telomeric DNA.

**FIGURE 5:**
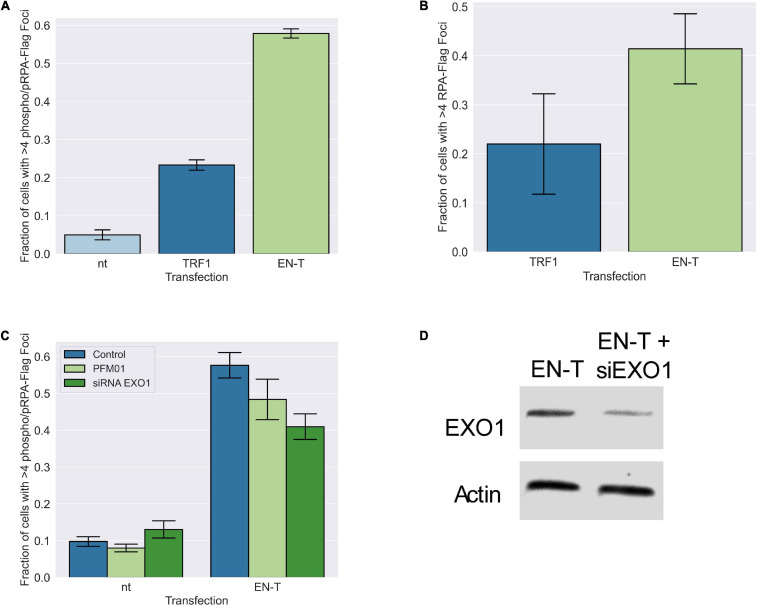
Replication protein A (RPA)-coated single-stranded DNA (ssDNA) at telomeric DSBs in BJ1-hTERT G1 cells is not significantly influenced by MRE11 or EXO1 nuclease activity. **(A)** Transfection of BJ1-hTERT cells with EN-T induced both phospo-RPA32 and **(B)** RPA70 foci in G1 cells that co-localized with EN-T (phospho RPA-32 *p* = 0.0000046, RPA70 *p* = 0.27). **(C)** Phospho-RPA32 induction following EN-T transfection was not significantly reduced by either inhibition of MRE11 endonuclease activity (PFM01), or siRNA knockdown of EXO1 (*p* = 0.24, 0.10, respectively). **(D)** siRNA knockdown of EXO1. All data represent three independent experiments (*n* = 30). Error bars are SEM, significance was established using ANOVA and *post hoc* Tukey’s HSD; *p* < 0.05 is significant.

To further validate the presence of ssDNA in cells transfected with EN-T, we immunostained for RPA70 and phospho-RPA32 (S4/S8). Following induction of telomere-specific DSBs, phospho-RPA32 showed pronounced and frequent colocalization with EN-T in BJ1-hTERT G1 cells (*p* = 0.0000046 Figure 5A); RPA70 foci were not significantly increased by expression of EN-T (*p* = 0.27 Figure 5B). Similar increases in (ss)telomeric DNA and phospho-RPA32 in EJ-30 G1 cells expressing EN-T were observed; as expected, this increase was also seen in S/G2 EJ-30 cells expressing EN-T (Supplementary Figure 5B).

### 5′ C-Rich Single-Stranded DNA at Telomere-Specific Double-Strand Breaks in G1 Is Not Dependent on Conventional Exonucleases, nor Does It Engage in Homology Dependent Repair

The presence of extensive tracks of ssDNA at telomeric DSBs in G1 suggested that long-range resection was occurring. Therefore, we investigated the role of conventional end-processing exonucleases MRE11 (3′-to-5′) and EXO1 (5′-to-3′), known mediators of resection at genomic DSB sites and at telomeres, respectively, in phospho-RPA foci induction following EN-T expression. Chemical inhibition of MRE11 (via treatment with the small molecule inhibitor PFM01) in EN-T expressing BJ1-hTERT G1 cells, did not significantly influence phospho-RPA32 foci, which were only slightly reduced compared with EN-T controls (*p* = 0.24) (Figure 5C). Phospho-RPA32 foci were also only slightly reduced when BJ1-hTERT cells were partially depleted of EXO1 (via siRNA); the difference was not statistically significant (*p* = 0.10) (Figures 5C,D). Thus, the majority of resection observed at telomeric DSBs in G1 human cells appears to occur independent of conventional resection machinery.

An alternative explanation for the presence of ssDNA at telomeric DSBs in G1 could be that it represents an attempt to regenerate a normal (ss)telomeric G-rich overhang for T-loop formation and end protection (Griffith et al., 1999). The SNM1B/Apollo (5′-to-3′) exonuclease has been shown to be necessary for generation of the telomeric 3′ G-rich overhang at blunt-ended leading-strand telomeres in mice (Wu et al., 2010, 2012). Therefore, we hypothesized that Apollo may act bidirectionally at telomeric DSBs, explaining the C-rich overhangs observed. However, EN-T-expressing Apollo^–/–^ EJ-30 human cells exhibited a slight reduction in C-rich (ss)telomeric foci in G1 compared with wild type (WT) cells (*p* = 0.37, Supplementary Figure 5A). Additionally, EN-T expressing Apollo^–/–^ EJ-30 cells in G1 displayed more telomere phospho-RPA32 foci than EN-T expressing EJ-30 G1 wild type cells (*p* = 0.099) (Supplementary Figure 5B). Furthermore, both measures of telomeric ssDNA were slightly increased in the Apollo^–/–^ S/G2 populations compared with wild type cells. Thus, the Apollo nuclease is not responsible for the extensive resection observed at telomeric DSBs in G1 human cells.

**FIGURE 6:**
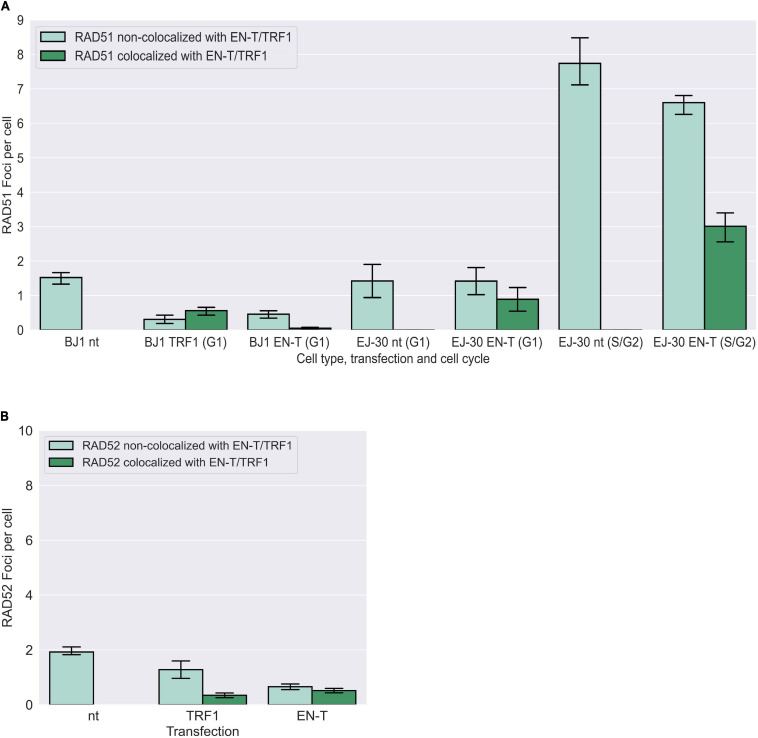
Homologous recombination (HR) not occurring at telomeric DSBs in G1. **(A)** Transfection of BJ1-hTERT or EJ-30 cells with EN-T did not induce RAD51 foci in G1. RAD51 foci were increased in S/G2 EJ-30 cells following expression of EN-T, consistent with HR activity during these phases of the cell cycle. **(B)** Transfection of BJ1-hTERT cells with EN-T also did not induce RAD52 foci in G1. All data represent three independent experiments (*n* = 30 BJ1 hTERT; *n* = 300 EJ-30 cells/experiment). Error bars are SEM, significance was established using ANOVA and *post hoc* Tukey’s HSD; *p* < 0.05 is significant.

To determine whether telomere DSB-induced resected (ss)telomeric DNA in G1 could represent an early element of HR-dependent repair (for strand-invasion), we also evaluated induction of RAD51 foci post EN-T transfection. While RAD51 foci were observed at telomeres in EN-T expressing EJ-30 S/G2 cells, they were *not* detected in EN-T expressing BJ1-hTERT or EJ-30 G1 cells (Figure 6A). Additionally, neither RAD52, nor repair-associated DNA synthesis (BrdU incorporation) were detected following EN-T induction of telomeric DSBs in BJ1-hTERT G1 cells (Figure 6B; Supplementary Figure 3C). Taken together, these results demonstrate that telomeric ssDNA at telomeric DSBs in G1 human cells is not generated by conventional DSB or telomere resection machinery, nor does it engage in resection-dependent recombinational repair, findings consistent with the majority of telomeric ssDNA in G1 being 5′ C-rich.

### 5′ C-Rich Single-Stranded Telomeric DNA at Telomere-Specific Double-Strand Breaks in Human Alternative Lengthening of Telomeres G1 Cells Is Bound by Telomere Repeat-Containing RNA, TERRA

Telomeric C-rich (ss)overhangs [5′-CCCTAA-3′] are a previously proposed marker of the recombination-dependent ALT pathway (Oganesian and Karlseder, 2011). Therefore, we evaluated whether EN-T induced telomeric DSBs in human U2OS (ALT) cells (Supplementary Figure 2) resulted in significant increases in 5′ C-rich (ss)telomeric DNA in G1. Considering the complementary nature of telomeric RNA, TERRA [5′-UUAGGG-3′], and that ALT cells possess higher levels of TERRA than non-ALT cells, we also monitored TERRA distribution, specifically in U2OS G1 cells. A stable U2OS cell line expressing Geminin protein fused to green fluorescence protein (Geminin-GFP) (Sakaue-Sawano et al., 2008) was generated to positively identify, and eliminate from analyses, cells in G2; CENP-F staining also confirmed that Geminin-GFP-positive cells were in G2. Cells negative for Geminin-GFP were in G1.

**FIGURE 7:**
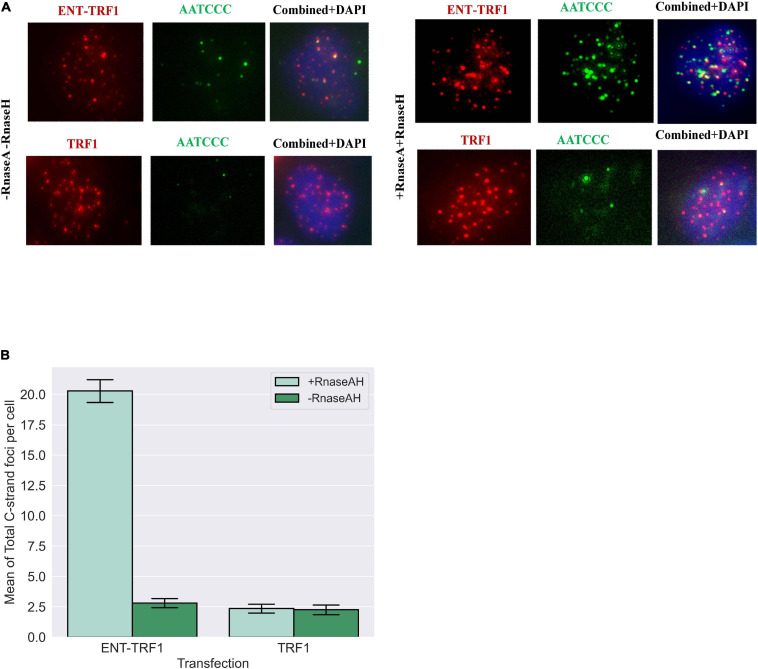
Telomere repeat-containing RNA (TERRA) accumulates at telomeric 5′ C-rich (ss)overhangs in G1 U2OS cells. **(A)** Representative images of FUCCI-U2OS G1 cells transiently transfected with EN-T or TRF1-only, labeled with the G-rich telomere probe to detect C-rich (ss)telomeric DNA (3′-AATCCC-5′), and merged views. Treatment with RNAseA and RNAseH removed telomeric RNA (TERRA) and revealed significant increases in complementary 5′ C-rich (ss)telomeric DNA. **(B)** Quantification of average number of C-rich telomeric foci per cell. All data represents three experiments and values are expressed as SEM (*n* = 120–200). Error bars are SEM, significance was established using ANOVA and *post hoc* Tukey’s HSD; *p* < 0.05 is significant.

Utilizing native (non-denaturing) DNA FISH to detect 5′ C-rich (ss)telomeric DNA, no enrichment in EN-T and TRF1-only transfected FUCCI-U2OS G1 cells was observed (Figure 7). However, treatment with RnaseA and RnaseH (to remove TERRA) revealed a highly significant increase in resected (ss)telomeric C-rich DNA in cells transfected with EN-T, but not with TRF1-only. Together, these results demonstrate telomeric RNA (TERRA) binding of 5′ C-rich (ss)telomeric DNA at telomeric DSB sites in G1 human ALT cells.

## Discussion

Telomere-specific DSBs have generally been regarded as irreparable, as DDRs generated globally by ionizing radiation or other genotoxic agents fail to resolve when they occur at or near telomeres and cells become senescent (Fumagalli et al., 2012; Hewitt et al., 2012). While repair of targeted telomeric DSBs has been observed in cycling cell populations, as well as specifically in S-phase, there is a dearth of evidence for DDRs or repair activity at telomeric DSBs in G1 cells (Doksani and De Lange, 2016; Mao et al., 2016). To better understand human cellular responses to telomeric DSBs in G1, we investigated enzymatically induced (EN-T) telomere-targeted DSBs, specifically in telomerase-positive BJ1-hTERT (normal) and EJ-30 (bladder cancer) cells, and in U2OS (ALT, osteosarcoma) cells.

Telomeric DSBs in G1 elicited early signatures of a DDR, as evidenced by γ-H2AX and MDC1 recruitment to telomere break sites. Notably, however, while the DDR biomarker 53BP1 was recruited to telomeric DSBs in S/G2–it was not present at those occurring in G1 cells. Functionally, 53BP1 is most often associated with c-NHEJ, where it regulates 5′-to-3′ end-resection, but 53BP1 can also partially restrict resection during alt-NHEJ and HR repair (Xiong et al., 2015; Ochs et al., 2016).

To gain mechanistic insight into this unexpected finding, we explored whether components of the telomere end-protection complex shelterin (De Lange, 2005, 2010) might be involved in thwarting 53BP1 recruitment to telomeric DSBs in G1 human cells. Telomere end-protection function was manipulated, without completely disrupting it, in an effort to alleviate inhibition of 53BP1 recruitment to telomeric DSBs while also avoiding dysfunctional telomere-induced foci (TIFs) (Cesare et al., 2013). As near complete siRNA knockdown of TRF2 is necessary for a TIF response, we utilized an siRNA sequence that resulted in partial, sub-TIF-inducing depletion of TRF2, and combined it with EN-T or TRF1-only transfection in BJ1-hTERT cells. While partial TRF2 knockdown did not result in a TIF response in untransfected cells, it also did not alleviate inhibition of 53BP1 recruitment to telomeric DSBs in transfected G1 cells. Depletion of TRF2 in EN-T transfected EJ-30 cells also did not affect telomere fragmentation, indicating that TRF2 does not impact telomeric DSB repair. The absence of 53BP1 at telomeric DSBs in G1 was also observed in EN-T-transfected human cells depleted of another potential candidate, DNA-PKcs, previously shown to play a role in mammalian telomere end protection (Bailey et al., 1999), and proposed to act in concert with TRF2 in preventing both c-NHEJ and alt-NHEJ at functional telomeres (Bombarde et al., 2010).

Compaction of telomeric chromatin has been proposed as a unifying physical mechanism by which shelterin protects telomeres from repair (Baker et al., 2011; Bandaria et al., 2016). Therefore, we tested whether decompaction of genomic DNA could alleviate the repression of 53BP1 recruitment to telomeric DSBs in G1 human cells. Similar to partial TRF2 knockdown, treatment of EN-T-transfected cells with a histone deacetylase inhibitor failed to result in recruitment of 53BP1 to telomeric DSB sites in G1, suggesting that it may not be possible to relieve any potential influence of shelterin-mediated end-protection on inhibition of 53BP1 recruitment to telomeric DSBs in G1 without full deprotection of telomeres.

Consistent with the lack of 53BP1 at telomeric DSBs in G1 human cells, no evidence of c-NHEJ was observed, as neither shRNA depletion of DNA-PKcs nor chemical inhibition of DNA-PKcs catalytic activity influenced the response to EN-T-induced telomeric DSBs. Considering that both 53BP1 and c-NHEJ impede DSB repair associated DNA resection, we hypothesized that telomeric DSBs in G1 human cells may be especially vulnerable to resection. Indeed, pRPA coated (ss)telomeric DNA was detected following EN-T-mediated induction of telomeric DSBs in telomerase-positive G1 cells, indicative of extensive resection at break sites, as the detection limit of FISH is on the order of 0.5 kb (Ramakrishnan and Sulochana, 2012). Importantly and consistent with rapid truncation events and overall telomere shortening, telomeric DSBs in G1 human cells facilitated formation and enrichment of 5′ C-rich (ss)telomeric DNA, an observation supported by minimal dependence on MRE11, EXO1, or Apollo exonucleases. Given the abundance of (ss)telomeric DNA at broken telomeres in G1, a potential role for resection- and/or replication-dependent repair was also interrogated; however, no evidence of RAD51 (HR/BIR), RAD52 (BIR/SSA), or BrdU incorporation was observed. Alt-NHEJ was also not a likely candidate for telomeric DSB repair in G1, since it utilizes only a few base pairs of homology (∼20) (Symington and Gautier, 2011; Truong et al., 2013) and is hindered by RPA binding to ssDNA (Deng et al., 2014).

Thus, although telomeric DSBs in G1 undergo extensive resection, they do not appear to be repaired in G1. One option may be that they attempt to reconstruct a 3′ G-rich (ss)overhang in order to form a protective T-loop and avoid c-NHEJ-mediated telomere–telomere fusion, and indeed, an increase in ss G-rich telomeric DNA at broken telomeres in G1 was observed. Interestingly, both 3′ G-rich and 5′ C-rich telomeric overhangs have been proposed to mediate T-loop formation (Verdun and Karlseder, 2006; Oganesian and Karlseder, 2011). Therefore, resection may serve to stabilize broken telomeres during G1. This idea is supported by the fact that naturally shortened telomeres do not undergo fusion until nearly all telomeric repeats have been lost, suggesting that telomeres of nearly any length can be protected from repair activity (Capper et al., 2007). Furthermore, (ss)telomeric overhangs at functional telomeres have been implicated in protection from repair (Gong and De Lange, 2010). To extend this line of reasoning, resected telomeric DSBs may simply persist into S/G2, where telomeres critically shortened and/or rendered dysfunctional by internal DSBs could be elongated via telomerase-mediated or recombination-based ALT mechanisms, a notion consistent with telomeric 5′ C-rich (ss)overhangs as markers of the ALT pathway of telomere length maintenance (Oganesian and Karlseder, 2011, 2013).

It also remains possible that some presently unappreciated pathway of repair operates at telomeric DSBs in human G1 cells. Potential candidates include RAD52-independent single-strand annealing (SSA), as SSA was shown to take place in RAD52^–/–^ cells (Kan et al., 2017). Importantly however, RNA-templated DSB repair has recently been reported in human cells, a pathway that would be resection dependent and potentially not mediated by other conventional repair factors (Meers et al., 2016; Mazina et al., 2017). Our finding of telomeric RNA/TERRA-bound 5′ C-rich (ss)telomeric DNA at telomeric DSB break sites in human ALT G1 cells is particularly enlightening in this regard. We propose that while resected telomeric 5′ C-rich (ss)overhangs at telomeric DSBs in human telomerase-positive G1 cells (with low levels of TERRA) are coated primarily with RPA, presumably to further hamper NHEJ, in ALT G1 cells (with higher levels of TERRA), transient telomeric RNA:DNA hybrids rapidly form to protect these exposed overhangs (Ohle et al., 2016). Such dynamic interactions serve to preserve telomeric 5′ C-rich (ss)overhangs at telomeric DSB sites so that they persist into S/G2 phase (D’Alessandro et al., 2018) for replication (telomerase-mediated) or HR-dependent (ALT) elongation as a means of repair and restoration of functional telomeres. Our results highlight the remarkable adaptability of telomeres, and so have important implications for chronic telomeric DNA damage, such as occurs in extreme environments (e.g., during long-duration spaceflight), in normal human G1/G0 cells with very low levels of telomerase (Luxton et al., 2020a,b), as well as for therapeutically relevant targets for disrupting telomere function and improving treatment of ALT-positive tumors.

## Data Availability Statement

The datasets presented in this study can be found in online repositories. The names of the repository/repositories and accession number(s) can be found below: The datasets generated and analyzed for this study can be found at https://github.com/Jared-Luxton.

## Author Contributions

CN, TA, DM, JM, and SB conceived the studies reported here. CN, TA, LT, DM, KM, and JL participated in sample collection, processing, experimental execution, and/or data processing and analyses. All authors analyzed the data, exchanged ideas, and edited the manuscript.

## Conflict of Interest

The authors declare that the research was conducted in the absence of any commercial or financial relationships that could be construed as a potential conflict of interest.

## Supplementary Material

Supplementary Figure 1 Characterization of telomere-specific cutting by endonuclease TRAS-ENT (EN-T). **(A)** Overexpressed EN-T or TRF1-only co-localized with telomere repeats in U20S, EJ-30, and BJ1 hTERT cells (shown). **(B)** Expression of EN-T in EJ-30 cells activated DDR signaling, evidenced by P-S1981-ATM and P-Thr68-CHK2. **(C)** Consistent with telomere-specific cutting, expression of EN-T in EJ-30 cells also resulted in fragmentation of telomeric DNA on southern blot of telomeric restriction fragments (TRF); **(D)** quantification of mean telomere length (TRFs) in non-transfected (nt), TRF-1 control, and EN-T transfected cells. Data represent three independent experiments, with *n* = 50 (U2OS), *n* = 30 (BJ1 hTERT) or *n* = 300 (EJ-30) cells/experiment. Error bars are SEM, *p*-values < 0.05 are significant.

Supplementary Figure 2 Additional characterization of EN-T system. **(A)** Transfection of *cycling* U20S (ALT) cells with EN-T triggered a telomeric DDR in terms of γ-H2AX foci, which frequently overlapped with EN-T. **(B)** EN-T induced telomeric DSBs in *cycling* U20S cells also stimulated recruitment of RAD51, and **(C)** RAD52, mediators of HR and BIR respectively, both of which frequently overlapped with ENT.

Supplementary Figure 3 DAPI intensity histograms for identifying cells in G1. **(A)** DAPI intensity histograms were generated from images (63x) of approximately 300 cells per experiment. Exclusion of Cyclin A from the low DAPI intensity peak region of the histogram (blue bars) verified that these cells were in G1 phase of the cell cycle; data shown represent merged histograms from 3 replicates totaling 300 EJ-30 cells. **(B)** DAPI intensity histograms were not necessary for identification of BJ1-hTERT G1 cells, as EN-T and TRF1-only transfected cells were almost exclusively negative for Cyclin A, consistent with the vast majority of transfected BJ1 hTERT cells being in G1 phase 48 h post transfection when analyses were done. Image illustrates that while the population of cells contains many cyclin A positive cells (red), the relatively few transfected cells (green foci; EN-T) were always cyclin A negative (in G1). **(C)** Additionally, BrdU incorporation was not detected in BJ hTERT cells transfected with EN-T, additional confirmation that cells were in G1.

Supplementary Figure 4 Compromised telomeric end-capping does not promote 53BP1 recruitment to broken telomeres. **(A)** Relaxation of chromatin via treatment with trichostatin A (TSA) did not result in 53BP1 foci induction in EN-T expressing cells at any concentration. **(B)** Partial depletion of TRF2 (siRNA knockdown) did not influence induction of 53BP1 foci in EN-T or TRF1-only transfected BJ1-hTERT cells. **(C)** siRNA knockdown of TRF2 also had no measurable effect on telomere length (Telomere Restriction Fragments; TRF) in EJ-30 cells transfected with EN-T. **(D)** Stable shRNA knockdown of DNA-PKcs did not promote 53BP1 recruitment to telomeric DSBs in EN-T transfected BJ1-hTERT cells.

Supplementary Figure 5 Apollo endonuclease is not responsible for extensive resection at telomeric DSBs. **(A)** Telomeric ssDNA (5′-CCCTAA-3′) was slightly reduced in EN-T expressing EJ-30 Apollo^–/–^ G1 cells relative to EN-T expressing control (wild type) EJ-30 cells (*p* = 0.37), and **(B)** phospho-RPA32 foci were increased (*p* = 0.099). Additionally, both telomeric ssDNA and phospho-RPA32 foci were increased in EN-T expressing EJ-30 Apollo^–/–^ S/G2 cells.
