# Supplementary figures and images for: Telomeric Double Strand Breaks in G1 Human Cells Facilitate Formation of 5′ C-Rich Overhangs and Recruitment of TERRA
Source: Front Genet. 2021 Mar 25;12:644803. doi: 10.3389/fgene.2021.644803 (PMC8027502; doi:10.3389/fgene.2021.644803)

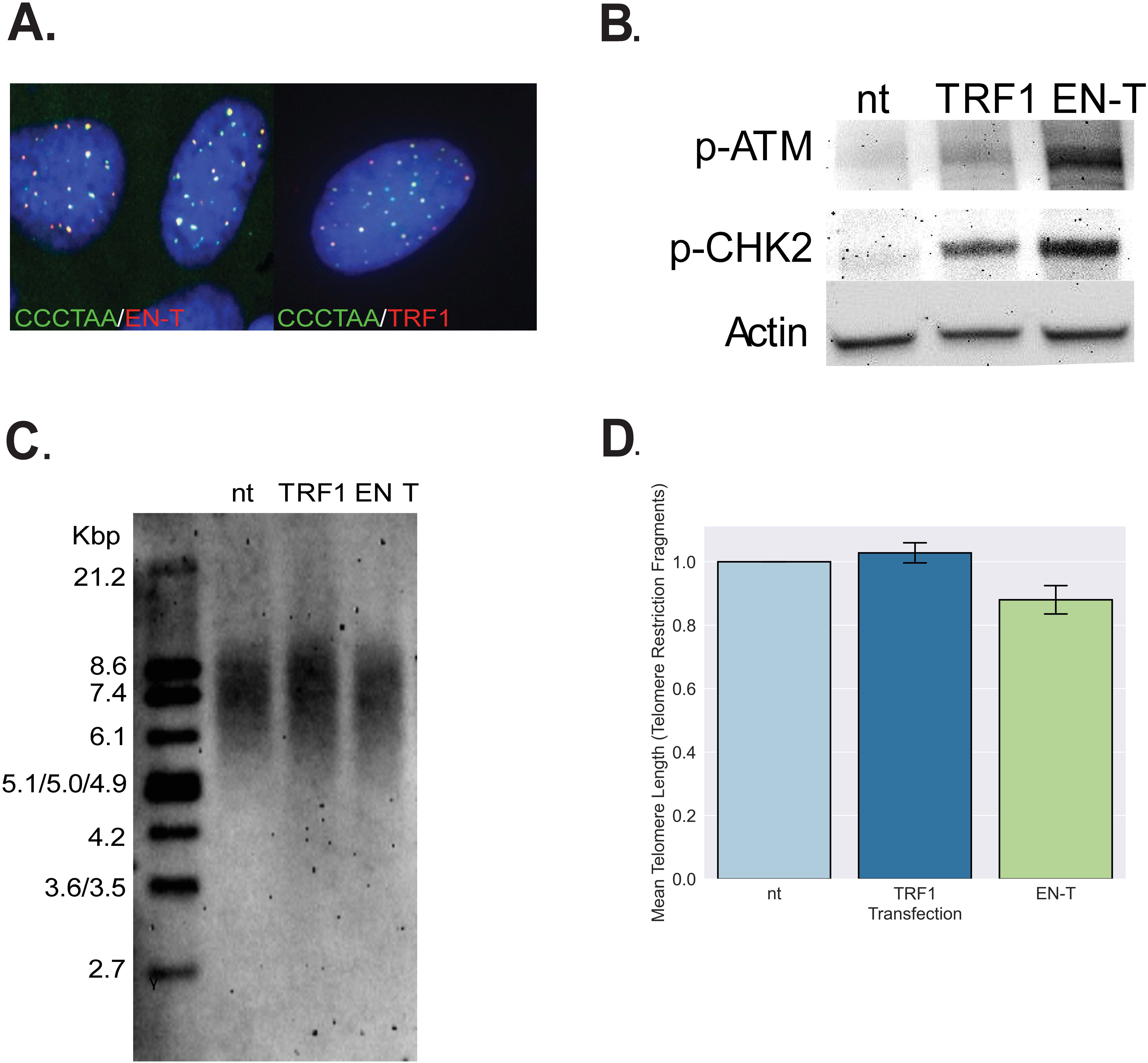

Supplement: Supplementary Figure 1 — Characterization of telomere-specific cutting by endonuclease TRAS-ENT (EN-T). (A) Overexpressed EN-T or TRF1-only co-localized with telomere repeats in U20S, EJ-30, and BJ1 hTERT cells (shown). (B) Expression of EN-T in EJ-30 cells activated DDR signaling, evidenced by P-S1981-ATM and P-Thr68-CHK2. (C) Consistent with telomere-specific cutting, expression of EN-T in EJ-30 cells also resulted in fragmentation of telomeric DNA on southern blot of telomeric restriction fragments (TRF); (D) quantification of mean telomere length (TRFs) in non-transfected (nt), TRF-1 control, and EN-T transfected cells. Data represent three independent experiments, with n = 50 (U2OS), n = 30 (BJ1 hTERT) or n = 300 (EJ-30) cells/experiment. Error bars are SEM, p-values < 0.05 are significant. [file Image_1.JPEG]

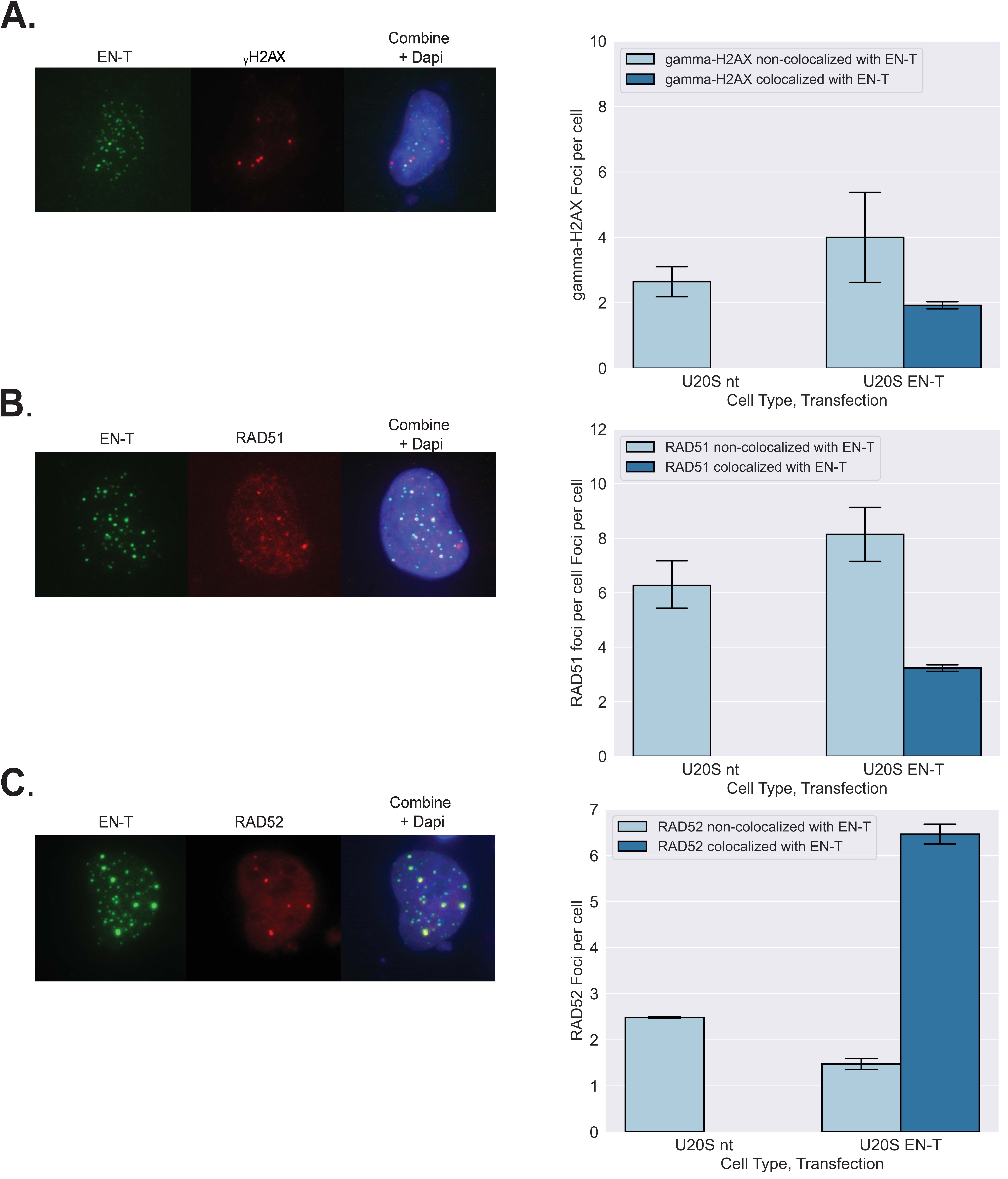

Supplement: Supplementary Figure 2 — Additional characterization of EN-T system. (A) Transfection of cycling U20S (ALT) cells with EN-T triggered a telomeric DDR in terms of γ-H2AX foci, which frequently overlapped with EN-T. (B) EN-T induced telomeric DSBs in cycling U20S cells also stimulated recruitment of RAD51, and (C) RAD52, mediators of HR and BIR respectively, both of which frequently overlapped with ENT. [file Image_2.JPEG]

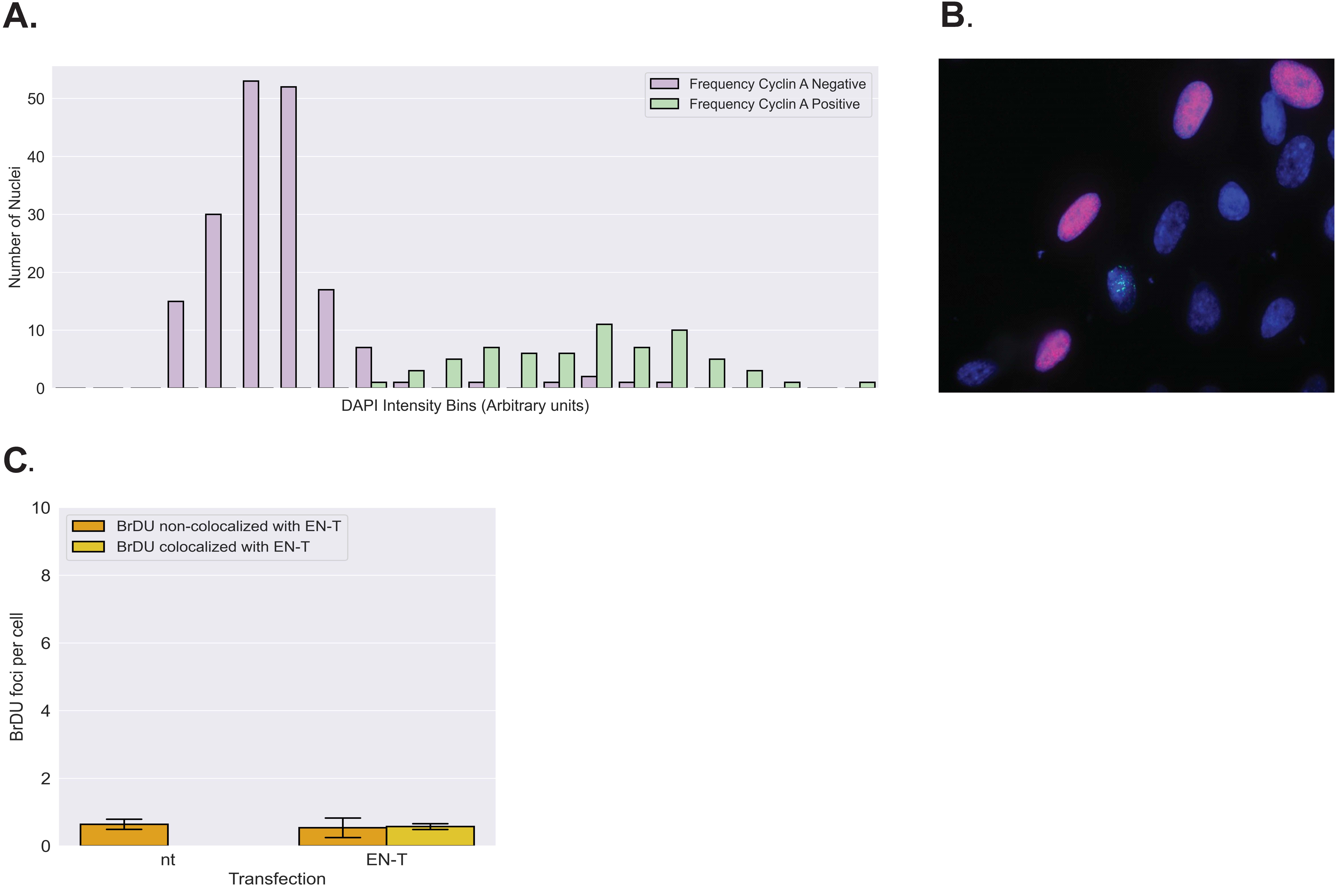

Supplement: Supplementary Figure 3 — DAPI intensity histograms for identifying cells in G1. (A) DAPI intensity histograms were generated from images (63x) of approximately 300 cells per experiment. Exclusion of Cyclin A from the low DAPI intensity peak region of the histogram (blue bars) verified that these cells were in G1 phase of the cell cycle; data shown represent merged histograms from 3 replicates totaling 300 EJ-30 cells. (B) DAPI intensity histograms were not necessary for identification of BJ1-hTERT G1 cells, as EN-T and TRF1-only transfected cells were almost exclusively negative for Cyclin A, consistent with the vast majority of transfected BJ1 hTERT cells being in G1 phase 48 h post transfection when analyses were done. Image illustrates that while the population of cells contains many cyclin A positive cells (red), the relatively few transfected cells (green foci; EN-T) were always cyclin A negative (in G1). (C) Additionally, BrdU incorporation was not detected in BJ hTERT cells transfected with EN-T, additional confirmation that cells were in G1. [file Image_3.JPEG]

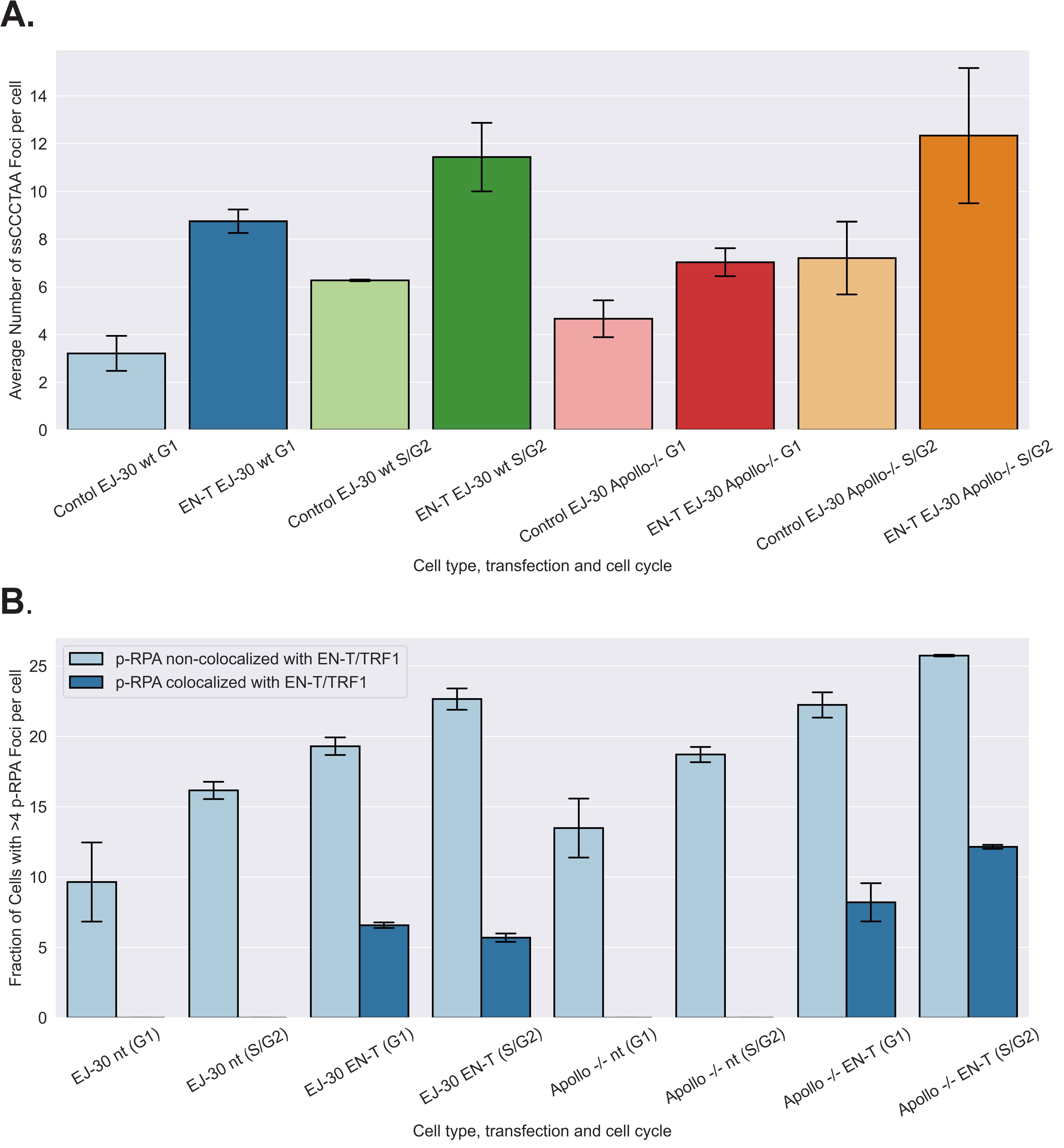

Supplement: Supplementary Figure 5 — Apollo endonuclease is not responsible for extensive resection at telomeric DSBs. (A) Telomeric ssDNA (5′-CCCTAA-3′) was slightly reduced in EN-T expressing EJ-30 Apollo–/– G1 cells relative to EN-T expressing control (wild type) EJ-30 cells (p = 0.37), and (B) phospho-RPA32 foci were increased (p = 0.099). Additionally, both telomeric ssDNA and phospho-RPA32 foci were increased in EN-T expressing EJ-30 Apollo–/– S/G2 cells. [file Image_5.JPEG]
